# A Comprehensive Review of Ethnomedicinal Uses, Phytochemistry, Pharmacology, and Toxicity of *Prunus africana* (Hook. F.) Kalkman from Africa

**DOI:** 10.1155/2024/8862996

**Published:** 2024-04-16

**Authors:** James K. Ndung'u, Joseph M. Nguta, Isaac M. Mapenay, Gervason A. Moriasi

**Affiliations:** ^1^Department of Public Health, Pharmacology, and Toxicology, University of Nairobi, P.O. Box 29053-00625, Nairobi, Kenya; ^2^Department of Pharmacy, Kenya Medical Training College, Nakuru Campus Kenya, P.O. Box 110, Nakuru, Kenya; ^3^Department of Biochemistry, Microbiology and Biotechnology, Kenyatta University, P.O. Box 43844-00100-GPO, Nairobi, Kenya; ^4^Department of Medical Biochemistry, Mount Kenya University, P.O. Box 342-01000, Thika, Kenya

## Abstract

*Prunus africana*, a widely utilized medicinal plant in various African ethnic communities, continues to hold significant importance in traditional healing practices. Research has identified phytochemical compounds in this plant, exhibiting diverse pharmacological activities that offer potential for pharmaceutical development. Notably, *P. africana* is employed in treating various ailments such as wounds, diabetes mellitus, malaria, benign prostatic hyperplasia, chest pain, and prostate cancer. Its pharmacological properties are attributed to a spectrum of bioactive compounds, including tannins, saponins, alkaloids, flavonoids, terpenoids, phytosterols, and fatty acids. Multiple studies have documented the anti-inflammatory, antimicrobial, antiandrogenic, antiangiogenic, antioxidant, antidipeptidyl peptidase-4 activity, analgesic, and astringent properties of *P. africana* extracts. This review offers a comprehensive compilation of ethnomedicinal applications, phytochemical composition, pharmacological effects, and toxicity assessments of *P. africana*, serving as a foundation for future preclinical and clinical investigations. By understanding its traditional uses and chemical constituents, researchers can target specific medical conditions with greater precision, potentially expediting the development of safe and effective pharmaceuticals. Moreover, toxicity assessments provide crucial insights into the safety profile of *P. africana* extracts, ensuring the development of safe pharmaceuticals to treat various diseases.

## 1. Introduction

In many developing regions, especially in sub-Saharan Africa, traditional medicine practitioners are the primary healthcare providers, due to their long-standing, often hereditary, ethnomedicinal knowledge about indigenous plants and their utility in treating diseases [1, 2]. The World Health Organization (WHO) notes that globally, many different plant species are utilized by various communities to treat various diseases [3]. The international focus on researching, recognizing, supporting, and promoting alternative and complementary medicine is expanding, due to an increasing need for efficacious and safe therapeutic alternatives for combating infections and diseases [4]. However, herbal medicines trade practices often jeopardize the sustainability of natural resources, as they are overexploited with limited conservation [5, 6]. The renewed enthusiasm for natural products as alternative medicines is attributed to concerns about conventional medicines, such as high costs, and adverse effects, limiting their clinical usefulness [7, 8]. In addition, the heavy global burden of disease coupled with the emergence of antimicrobial resistance further threatens public health and highlights the need for substitute medications, particularly those derived from natural sources [9–11].

*Prunus africana* (Hook. F.) Kalkman, commonly known as African cherry, African plum, African prune, or bitter almond, belongs to the Rosaceae family, subfamily Amygdaloideae syn. Prunoideae, and subgenus *Laurocerasus* [12, 13]. The term “Prunus” denotes the plum-like shape of its fruit, and “africana” signifies its endemic existence in the Afromontane forests where locals utilize it for various medicinal and household purposes [13, 14]. *Prunus africana* is commonly used to treat benign prostatic hyperplasia (BPH) and has been reported to contain various secondary metabolites with anticancer, analgesic, anti-inflammatory, antimicrobial, and antiviral activities [15, 16]. It is also utilized in traditional medicine to manage many ailments such as malaria, fevers, mental illness, and gastrointestinal disorders [14–16].

Performing an in-depth review of *Prunus africana* and highlighting its biological activities, toxicity, and phytochemical components is crucial as it provides an understanding of the therapeutic benefits and safety. This review consolidates and provides important insights into its ethnopharmacology, phytochemistry, and bioactivity to guide future research and drug development endeavours.

## 2. Methodology

### 2.1. Search Strategy

We conducted a thorough search for relevant information by exploring electronic literature databases, including PubMed, Web of Science, Scopus, Science Direct, US National Library of Medicine, Cochrane Library, and Google Scholar. The specific search terms included “*P. africana*,” “*African cherry*,” and “*Pygeum africanum*,” combined with connector terms such as “phytochemicals,” “traditional uses,” “ethnomedicine,” “biological activities,” and “toxicity/safety.” Additionally, searches were performed for specific topics such as “phytochemicals in *P. africana*,” “disease treatment,” “safety and toxicity,” “traditional medicine,” and “medicine.” Only articles in the English language were considered. This search initially yielded 467 articles.

### 2.2. Study Inclusion and Exclusion Criteria

This review included studies that met the following criteria: (i) published in English in peer-reviewed and internationally refereed journals; (ii) *in vitro* and *in vivo* experiments examining *P. africana* extracts or compounds with appropriate controls (positive and negative); (iii) documentation of traditional/ethnomedicinal uses of *P. africana*; and (iv) scientific reports of pharmacological/biological activities of extracts/isolated compounds from *P. africana*.

The exclusion criteria included the following: (i) studies whose primary focus was not *P. africana*; (ii) studies without data regarding traditional uses, phytochemistry, toxicity/safety, and pharmacological/biological effects; (iii) non-English language publications; and (iv) studies that did not have appropriate control groups. We used a systematic screening process to identify eligible studies for inclusion in our review, adhering strictly to these outlined criteria.

### 2.3. Assessment of Study Quality and Risk of Bias

**Figure 1:**
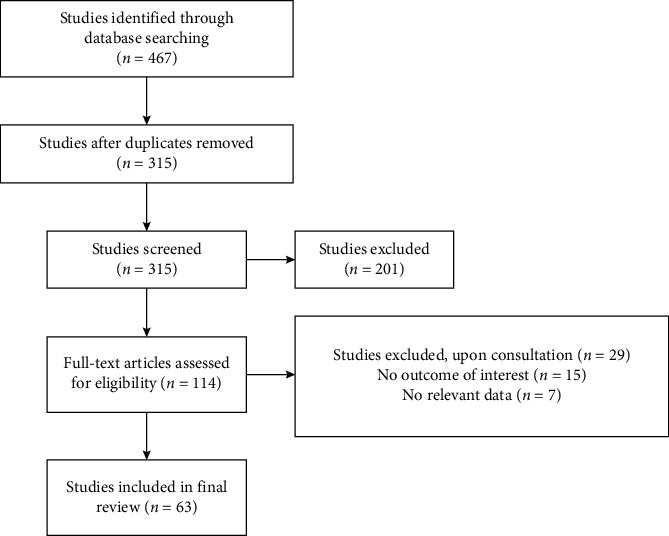
Schematic flowchart showing the search strategy and selection of eligible studies.

Study quality and risk of bias assessments were independently conducted by two investigators, James Ndung'u and Gervason Moriasi, utilizing previously described criteria [17,18]. Assessment parameters encompassed the completeness of outcome data, selective reporting, lack of appropriate control experiments, lack of randomization of study subjects, and other sources of bias. Discrepancies were resolved through consultation with the other two investigators, James Nguta and Isaac Mapenay. Data accuracy and appropriateness were ensured through verification and collaborative discussions among the authors. Following the verification process and removal of duplicate documents, 63 articles were deemed suitable for inclusion in this review (Figure 1).

## 3. Results and Discussion

### 3.1. Ethnomedicinal Uses

*Prunus africana* is distributed widely in Africa and is renowned for its medicinal properties across its various parts—leaves, roots, and stem bark [19] (Table 1). *P. africana* stem bark is a key component of treatment for gastrointestinal disorders, respiratory problems, benign prostatic hyperplasia (BPH), and chest pain in South Africa [14, 36]. Similarly, in East Africa, the plant's potential is harnessed for addressing acquired immunodeficiency syndrome (AIDS)-related symptoms, along with managing diverse health concerns such as BPH, respiratory ailments, diabetes, hypertension, kidney diseases, malaria, and prostate cancer [21, 23, 28].

Previous research by Stewart [14] and Kipkore et al. [28] supports the extensive use of *P. africana*'s roots, leaves, and fruits in East Africa to address various health issues. In West Africa, particularly Cameroon, the ethnomedicinal application of the plant's stem bark is notable in treating respiratory conditions, wound healing, mental health issues, malaria, prostate cancer, and chest pain [14, 20, 24].

The ethnomedicinal findings underscore the plant's remarkable versatility, spanning a spectrum of health conditions from gastrointestinal problems to prostate cancer, high blood pressure, digestive cleansing, skin infections, and beyond [14]. These applications not only attest to its efficacy but also illuminate its seamless integration into traditional medical practices, rendering it an invaluable resource [26, 28, 29, 37]. In addition, this botanical versatility underscores the rich pharmacological reservoir that *P. africana* represents in traditional African medicine. Its multifaceted application in treating various ailments highlights the profound empirical knowledge embedded in indigenous healing practices [38]. Moreover, the diversity of conditions it addresses underscores the potential for further exploration of its bioactive compounds and mechanisms of action, providing opportunities for novel drug development or complementary therapeutic approaches.

However, despite its widespread use and promising therapeutic indications, it is imperative to bridge the traditional and modern realms of medicine through rigorous scientific investigation [35]. Conducting comprehensive studies to elucidate the active constituents, pharmacological mechanisms, and safety profiles of *P. africana* extracts or derivatives can facilitate its integration into mainstream healthcare practices. Additionally, efforts to promote sustainable harvesting and conservation practices are essential to safeguard the ecological balance and ensure the long-term availability of this valuable botanical resource.

### 3.2. Phytochemistry

This review noted that various parts of *P. africana*, especially its stem bark, contain various secondary metabolites, as summarized in Table 2. The prominent phytochemicals include terpenoids, such as ursolic acid, oleanolic acid, and *β*-amyrins [16, 41, 42]. In addition, the stem bark of *P. africana* also contains flavonoids such as ferulic acid, phytosterols, prominently *β*-sitosterol, and *β*-sitostenone, and fatty acids, including lauric acid, and myristic acid [16, 41–45]. Moreover, Wavinya Nyamai et al. [16] and Komakech et al. [39] identified atraric acid and N-butylbenzene-sulfonamide as specific compounds representing tannins in *P. africana*'s bark.

This compilation provides a nuanced understanding of the diverse phytochemical constituents in the stem bark of *P. africana*, shedding light on its potential bioactive properties. The synergistic interactions among the bioactive phytochemicals present in *P. africana* underscore its profound efficacy as a traditional medicinal resource. These interactions have been observed to exert potent therapeutic effects across a wide spectrum of diseases and conditions [16, 37, 41, 46, 47]. The documented interplay of terpenoids, flavonoids, phytosterols, fatty acids, and tannins within the plant's composition highlights the multifaceted pharmacological potential that contributes to its traditional medicinal prowess [48].

Nonetheless, despite the significant progress made in understanding the phytochemical profile of *P. africana*, several gaps remain to be addressed in future studies. For instance, while individual compounds have been identified, further research is needed to elucidate the synergistic interactions among these constituents and their collective pharmacological effects. Additionally, more comprehensive pharmacological studies, including in vitro and in vivo experiments, are warranted to validate the therapeutic potential attributed to *P. africana* and its individual constituents. Moreover, investigations into the mechanisms of action underlying the observed bioactivities are crucial for a deeper understanding of its therapeutic effects. Furthermore, given the increasing demand for natural products in pharmaceuticals, studies focusing on sustainable cultivation, extraction methods, and conservation strategies for *P. africana* are essential to ensure its long-term availability and viability as a medicinal resource.

### 3.3. Biological Activities

Phytochemicals derived from *P. africana* exhibit a diverse array of biological activities, encompassing anticancer, anti-inflammatory, antimicrobial, and antiviral properties, as summarized in Table 3. Ursolic acid, oleanolic acid, and *β*-amyrins, prominent terpenoids found in *P. africana*, have demonstrated notable anticancer effects through various mechanisms, including the induction of apoptosis and inhibition of tumor cell proliferation [39, 41]. Furthermore, these compounds possess potent anti-inflammatory properties, making them promising candidates for the treatment of inflammatory conditions [16, 41, 50].

Additionally, *P. africana*-derived phytochemicals such as tannins and terpenoids have exhibited antimicrobial and antiviral activities, suggesting their potential in combating infectious diseases [23, 46, 53–56]. The elucidation of these biological activities not only underscores the therapeutic potential of *P. africana* but also highlights the importance of further research to explore their mechanisms of action and clinical applications. The reported biological activities of phytochemicals in *P. africana* offer valuable opportunities for the discovery and development of novel therapeutic agents [65, 66]. By elucidating the mechanisms underlying their anticancer, anti-inflammatory, antimicrobial, and antiviral effects, researchers can identify potential drug targets and develop pharmacological interventions with improved efficacy and safety profiles [7, 67]. Furthermore, the identification of synergistic interactions among different phytochemical constituents of *P. africana* may pave the way for the development of combination therapies with enhanced therapeutic outcomes [68, 69]. Moreover, the exploration of structure-activity relationships of these phytochemicals could facilitate the design and synthesis of analogues with improved bioavailability and pharmacokinetic properties [70]. Thus, leveraging the biological activities of phytochemicals in *P. africana* holds immense potential for the development of new therapeutic agents to address unmet medical needs.

Recognizing the therapeutic potential of *P. africana*-derived phytochemicals may prompt policymakers to support research initiatives aimed at exploring their pharmacological properties and clinical applications. Moreover, integrating evidence from preclinical and clinical studies into healthcare policies may facilitate the inclusion of *P. africana*-based interventions in clinical practice guidelines, thereby expanding treatment options for various diseases [71, 72]. Furthermore, promoting the sustainable cultivation and harvesting of *P. africana* may contribute to the conservation of biodiversity and support local economies in regions where this plant species is endemic [5]. Thus, evidence-based policymaking informed by research on the biological activities of *P. africana*-derived phytochemicals can have far-reaching implications for public health and environmental sustainability.

### 3.4. Antibenign Prostatic Hyperplasia (BPH)

About half of men who are 50 years of age or older have BPH, a nonmalignant enlargement of the prostate gland. Practitioners in traditional medicine have used *P. africana* bark decoction as an effective remedy for BPH for many years [19, 47]. The stem bark may be rendered into a decoction by powdering and boiling it in water, or alternatively, the powdered bark can be encapsulated and orally administered to manage and treat BPH [16]. Ethnobotanical surveys substantiate the prevalence of *P. africana* in managing BPH. Ethnomedicinal investigation reveals the significant utilization of *P. africana* in treating prostatic ailments within the Foumban community in Cameroon [73]. Likewise, a study by Kipkore et al. [28] highlights the Marakwet community in Kenya employing the decoction of *P. africana* stem bark for BPH treatment and management.

*In vitro* studies demonstrate that *P. africana* stem bark extract inhibits the proliferation of fibroblasts from human hyperplastic prostate and bladder, underscoring the plant's potential in BPH treatment [74]. Wavinya Nyamai et al. [16, 75] reported that in double-blind, placebo-controlled clinical trials, daily doses between 75 and 200 mg result in favorable outcomes: decreased prostate size, increased urine flow, decreased frequency of urination, and decreased irritative symptoms—all within a six- to three-month timeframe.

The effectiveness of *P. africana* in treating BPH has been reported by Jena et al. [74], Wavinya Nyamai et al. [16], and Letoyah [47]. These studies attribute this to the synergistic actions of various phytochemicals, including ferulic acid and pentacyclic triterpenoids like ursolic acid and phytosterols like *β*-sitosterone. By inhibiting the alpha-reductase enzyme, these compounds restore testosterone concentration, mitigate inflammatory symptoms, and exhibit antioxidant properties, resulting in anti-BPH efficacy [39].

Scientific investigations reveal *P. africana* extracts' potent antiandrogenic activity, impeding androgens' (e.g., testosterone and dihydrotestosterone) biological effects [39, 76]. Additionally, the high myristic acid content in *P. africana*'s stem bark exerts antioxidant effects, curtailing susceptibility to lipid peroxidation and averting oxidative stress, a key contributor to BPH [39, 59]. The pharmacological impact of *P. africana* phytochemicals on BPH validates and demystifies its ethnobotanical application in traditional medicine for BPH treatment and management. The convergence of ethnobotanical knowledge with scientific evidence underscores the pharmacological potential of *P. africana* in BPH treatment, bridging traditional medicine knowledge with modern therapeutic approaches.

### 3.5. Antiprostate Cancer

Roughly 15% of men have prostate cancer [77]. In recent decades, researchers have identified various plants, including *P. africana*, as reservoirs for effective chemopreventive and therapeutic agents against prostate cancer, among other cancers [39, 78, 79]. Extensive ethnobotanical evidence supporting the use of *P. africana* in treating and managing cancers, including prostate cancer, has been presented in the literature by various scholars [14, 22, 28]. Ochwang'i et al. [20] conducted a study and noted that traditional healers in Kakamega County, Kenya, use *P. africana* bark decoction can be used to treat cancers such as prostate cancer and other urological symptoms.

The use of *P. africana* decoction for the treatment of cancer and related conditions was confirmed by another survey conducted by Tugume et al. [31] close to the Mabira Central Forest Reserve in Uganda.

Several key phytochemicals (Table 3) in *P. africana* are responsible for its anticancer and antitumor properties. Notably, beta-sitosterol and ursolic acid demonstrate anti-inflammatory effects on the prostate gland, denoting its antiprostate cancer activity [16]. Furthermore, ferulic acid esters along with their derivatives actively contribute to both antitumor function and hypocholesterolemic activities of the *P. africana* bark extract on prostatic tissues [16, 39]. Asuzu et al. [58] and Komakech et al. [59] reported that ethanolic stem bark extract of *P. africana* stem bark significantly inhibited the growth of human prostate cancer cell lines (PC-3 and LNCaP) (IC_50_ : 2.5 *µ*l/ml), by inducing apoptosis compared to control cells. In vitro studies suggest a promising strategy that the antiprostate activity of *P. africana* is driven by ursolic acid, which downregulates B-cell lymphoma 2, thereby inducing apoptosis in PC-3 cells [59]. Additionally, its beta-sitosterol and ferulic acid trigger apoptosis not only in PC-3 cells but also in LNCaP human prostate cancer cells, demonstrating an expansive range of potentially impactful treatments at our disposal [16, 59].

Following a comprehensive review, Komakech et al. [39] concluded that *P. africana* holds substantial potential in chemoprevention and chemotherapy for prostate cancer—primarily because of its constituent phytochemicals (Table 3), such as atraric acid and N-butylbenzene-sulfonamide, which suppress the androgen receptor, thus reducing PC-3 proliferation [16, 59]. In other studies, it was demonstrated that oleanolic acid also suppresses the androgen receptor to avert PC-3 cells' proliferation [39, 58, 59, 80]. Furthermore, research shows lauric acid inhibits the 5-*α*-reductase enzyme, which blocks the conversion of testosterone to dihydrotestosterone, in turn, preventing prostate cancer [60, 81]. Moreover, these phytochemicals exert their antitumor activity by activating the 5′-AMP-activated protein kinase (AMPK) enzyme, disrupting key metabolic pathways in prostate cancer cells [55, 56].

The synergistic effects of the phytochemical compounds of *P. africana* lead to a reduction in cancer cell proliferation, early senescence, and senescence-associated beta-galactosidase activity [47, 82]. Ngule and Francis [46] and Letoyah [47] attributed these antiproliferative effects to tannins. Research has shown that tannins provide essential protection against oxidative damage—especially lipid peroxidation—due to their strong antioxidative properties, consequently functioning as potent anticarcinogenic agents with antimutagenic capabilities [83].

In addition, flavonoid polyphenols in *P. africana* [41, 47, 84] provide antioxidative protection against free radical damage, thereby ameliorating diseases such as cancer and diabetes [85]. The action of flavonoids involves the scavenging of free radicals, chelation of metal ions, especially those implicated in oxidative stress, and inhibition of enzymes responsible for generating these harmful entities [48, 86]. In a recent in vitro study conducted by Muruthi et al. [87], it was confirmed that plant-derived flavanols not only exhibit inhibitory effects on human cancer cells but also provide protection against the oxidation process of low-density lipoprotein. Nevertheless, these studies are not exhaustive, and more need to be done to ensure that these efficacies are translated into clinical practice.

### 3.6. Cognitive-Enhancing Effects

Recently, Ngai et al. [41] have investigated the cognitive-enhancing effects of the methanolic leaf and stem bark extracts of *P. africana* based on its ethnomedicinal application in managing mental illnesses [22]. The extracts exhibited a considerable anticholinesterase activity and improved cognitive function in scopolamine-induced cognitive-impaired Swiss albino mice [41]. These effects were attributed to the diverse phytochemicals such as quercetin, *β*-sitostenone, chlorogenic acid, apigenin, and ursolic acid, among others [16, 39, 41]. The proposed mechanisms of the bioactivity of these compounds included the inhibition of cholinesterase activity, the remediation of oxidative stress, and anti-neuroinflammation [39, 41]. Based on this report, these extracts may be potential sources of lead molecules for developing drugs to treat neurodegenerative diseases, such as AD.

The identification of phytochemicals with robust cognitive-enhancing properties underscores the importance of exploring natural products as reservoirs for drug development. The presence of compounds such as quercetin and ursolic acid, known for their neuroprotective effects, hints at the rich pharmacological diversity these extracts offer [16, 41]. Leveraging such bioactive compounds could lead to the development of innovative therapeutic interventions for neurodegenerative disorders, which continue to pose significant challenges in healthcare [86]. However, the journey from the natural extract to the clinical drug candidate is arduous and necessitates rigorous preclinical and clinical evaluations. Thus, while the study by Ngai et al. [41] provides a promising foundation, it also underscores the need for further in-depth investigations to elucidate the safety, efficacy, and mechanisms of action of these extracts. Collaborative efforts between traditional medicine practitioners, pharmacologists, and medicinal chemists are imperative to navigate the intricate path towards translating these natural remedies into clinically viable treatments for neurodegenerative diseases.

### 3.7. Antidiabetic Activity

The International Diabetes Foundation [88] and the World Health Organization [89] characterize diabetes as a complex and persistent medical condition, resulting from either the pancreas producing insufficient insulin or the body failing to utilize this hormone effectively leading to elevated levels of blood glucose. Among an array of herbal remedies found in local markets, *P. africana* has gained recognition for its potential in diabetic treatment [90]. Studies have shown that extracts from *P. africana* possess an intriguing ability to reduce the activity of dipeptidyl peptidase-4 enzyme (DPP-4), which plays a vital role in deactivating glucagon-like peptide (GLP-1), to increase insulin production. This is a pivotal approach in type 2 diabetes mellitus management and controlling and maintaining blood glucose levels [91–93], underscoring its potential efficacy.

In mitigating oxidative stress in the bladders of diabetic patients and decelerating the progression of diabetic cystopathy, preliminary interventions showed remarkable outcomes upon utilizing *P. africana*. After inducing diabetes in adult Wistar male rats for initial 4 weeks, orally administered 100 mg/kg of *P. africana* suspended in peanut oil produced suppression hyperglycaemia and diabetes-associated symptoms [94]. In the same study, the results demonstrated effectiveness in managing complications associated with diabetes in the rat, by not only reducing bladder-related issues but also addressing potential renal damage via its antioxidative properties. Furthermore, both aqueous and ethanolic extracts from *P. africana* showed noteworthy hypoglycaemic effects in alloxan-induced diabetic rats [95].

The observed antidiabetic properties of the *P. africana* stem bark are due to the presence of certain phytochemicals, notably: alkaloids, tannins, flavonoids, and saponins [46]. Tannins have a recognized role in diabetes treatment, as they reduce both plasma glucose and lipid profiles, hence significantly decreasing blood glucose levels without inducing adiposity [85, 96, 97]. Saponins also play an important part in their significant antidiabetic effects, as they lower blood glucose levels in diabetic patients according to previous reports [97, 98]. Flavonoids are responsible for battling diabetic complications, by modulating the effects on blood sugar transporters, enhancing insulin secretion—an action that also mitigates insulin resistance, and alleviating inflammation and oxidative stress in muscles [97–100]. Alkaloids in *P. africana* exhibit antidiabetic effects by targeting a variety of factors, thus leading to the attenuation of glucose-6-phosphatase, significantly reducing free glucose levels in the bloodstream [96]. Notably, Tiong et al. [101] presented compelling evidence from vitro studies that alkaloids hold considerable therapeutic promise against type 2 diabetes by facilitating glucose uptake by pancreatic beta-TC6 or myoblast C2 C12 cells. Although this review provides a persuasive account of the antidiabetic potential of *P. africana*, it is imperative to acknowledge that further research is needed to determine the efficacy of these extracts in human subjects and the clinical relevance of the reported findings.

### 3.8. Antimalarial Activity

Malaria stands as a prominent global health challenge, contributing to an annual death toll of one to two million individuals in Africa [21]. In response to this health burden, various African communities extensively leverage locally available medicinal plants for therapeutic purposes, with *P. africana* emerging as a cornerstone in malaria treatment [1, 19]. *P. africana's* role in malaria treatment has been previously underscored in a comprehensive study exploring the diversity and utilization of antimalarial ethnophytotherapeutic remedies among the Kikuyus in Central Kenya [21]. A parallel ethnomedicinal survey in Meru District, Kenya, corroborated the usage of *P. africana* in combating malaria [102, 103]. Typically, the stem bark powder is used to make an oral decoction or infusion that is used to treat malaria [104–106].

The efficacious attributes of *P. africana* in malaria treatment find scientific validation owing to its potent tannins, saponins, terpenoids, and alkaloids, constituting major antimalarial phytochemicals across a spectrum of plants used in Africa [107]. Tannins, recognized for their prophylactic potential, assume significance as bioactive chemicals with antimalarial properties [108]. Serge et al. [109] observed that alkaloid extracts from *P. africana* demonstrated a notable *in vitro* antiplasmodial activity against the *P. falciparum* strain, with an IC_50_ of 2.36 *µ*g/ml at a 24-hour incubation and 2.56 *µ*g/ml after 48 hours. Similarly, Lehane and Saliba [110] highlighted the *P. africana* flavonoids' inhibitory effect on the intraerythrocytic growth of P. falciparum in vitro, underscoring its potential effectiveness in treating malaria infections.

Rodrigues Goulart et al. [111] demonstrated that terpenoids of *P. africana* not only impeded *Plasmodium falciparum* but also suppressed isoprenoid biosynthesis. This finding's significance becomes even more apparent when we consider that P. falciparum relies heavily on isoprenoid synthesis for its survival during erythrocytic stages; this includes processes such as transfer ribonucleic acid (tRNA) isopentenylation and protein prenylation, as well as generating essential compounds such as vitamin E, carotenoids, dolichols, and ubiquinone [112]. Murata et al. [113] reported that terpenoids possess a potent inhibitory effect against various microbes, including *P. falciparum*, correspondingly suggesting that these compounds could be useful in combating parasitic infections. Moreover, terpenoids in *P. africana* extracts can hinder the synthesis of crucial biomolecules and, therefore, impede *P. falciparum* development [114]. Overall, the amalgamation of traditional use and scientific validation underscores the promising role of *P. africana* in the treatment and management of malaria, emphasizing its potential as a valuable resource in diverse African communities. However, further empirical investigations are required to determine the specific compounds, mechanisms of actions, and the translation of the in vitro results into clinical practice.

### 3.9. Management of Gastrointestinal Disorders

Drawing from human and animal models, a correlation between gastrointestinal disorders, such as stomach pain, and alterations in the microbiota resulting from consuming contaminated foods or water has been demystified with greater profundity [115–120]. Elsewhere, Stark et al. [121] documented the traditional African use of *P. africana* stem bark to treat diarrhea and abdominal conditions, highlighting the growing recognition in medical discourse that specific botanical remedies can address distinct ailments.

Ethnobotanical investigations in the south-west ethnoecological region of Cameroon by Jiofack et al. [24] revealed the use of *P. africana* bark decoction to address heartburn and gastralgia. Similarly, Amiri and Kisangau [122] reported the administration of *P. africana* bark decoction to alleviate stomachache in communities near the Kimboza Forest Reserve in Morogoro, Tanzania. Similarly, South African communities use the stem bark decoction of *P. africana* to treat gastric and abdominal conditions as reported by Eldeen et al. [123].

The efficacy of *P. africana* in managing gastrointestinal (GI) disorders is attributed to its potent antibacterial effects, providing protection against bacteria, a significant contributor to such ailments [123]. This claim was later supported by Chrispus Ngule et al. [124], who demonstrated robust antibacterial activities in the hydromethanolic stem bark extract of *P. africana*. Further examination revealed that tannins, found in *P. africana*, demonstrate inhibitory effects on the growth of microbial populations in various environments, including the human gastrointestinal tract [83, 125–127]. Based on this, Chrispus Ngule et al. [124] linked the presence of tannins in *P. africana* to astringency agreeing with earlier assertions by Ashok and Upadhyaya [128] that this is an important feature in preventing diarrhea and managing hemorrhage. Research indicates that these compounds achieve this by precipitating proteins and mucus as well as constricting blood vessels [126, 129, 130]. In addition, flavonoids may also alleviate both acute and chronic diarrhea through their ability to suppress intestinal motility and excessive mucus secretion, and their potential in alleviating the chronic inflammation of the gastrointestinal tract, by protecting against oxidative stress and preserving proper mucosal functioning [131]. Further research indicates that flavonoids exhibit remarkable therapeutic capabilities and can ameliorate inflammatory bowel disease effectively by preventing distressing symptoms such as bloody diarrhea and gastrointestinal pain [131]. De Lira Mota et al. [132] elucidated the pharmacological attributes of flavonoids in gastroprotection, antisecretory, cytoprotective, and antioxidant actions. These play a substantial role not only in treatment but also in controlling gastrointestinal disorders at large [131]. Further extensive studies aimed at identifying and characterizing specific compounds with the reported efficacies and establishing their mechanisms of bioactivity and safety are imperative to foster the translation of these results into clinical practice.

### 3.10. Antimicrobial Activity

Many communities utilize herbal remedies, renowned for potent antimicrobial properties, to manage skin infections, among other prevalent health maladies primarily attributable to bacteria, viruses, and fungi [19, 133, 134]. For instance, local communities around Nandi Forest in Kenya have for a long time utilized preparations derived from *P. africana* as topical applications for the treatment and mitigation of various adverse skin conditions, owing to the robust antifungal activity exhibited by the plant's tannins [19, 130]. Bii et al. [23] found that the methanolic stem bark extract of *P. africana* significantly combated dermatophytes, pathogenic fungi, that thrive on skin and other bodily surfaces to cause conditions such as ringworm—this aligns with traditional utilization of *P. africana* for its remarkable efficacy against these infections.

Research illuminates that tannins can hinder a wide range of microorganisms' growth including fungi, yeasts, viruses, and bacteria [83, 129]. In addition, Akiyama et al. [135] reported that galloyl catechins and ellagic acid derivatives derived from traditional Japanese medicines possess significant antimicrobial effects against these pathogenic agents. Mabhiza et al. [136] observed significant inhibitory effects of tannins and alkaloids on *Staphylococcus aureus* and *Pseudomonas aeruginosa* growth, which are a major cause of skin and soft tissue infections and were comparable to that of ampicillin (reference antibiotic). Henceforth, owing to its inherent antifungal and antibacterial properties—particularly in the stem bark where tannins are found—the phytochemicals within *P. africana* substantiate why diverse communities have utilized this plant for treating and managing various types of skin infections.

### 3.11. Chest Pain and Respiratory Conditions

African communities prevalently utilize a decoction derived from the bark of *Prunus africana* to treat and manage chest pain and respiratory ailments such as asthma, allergies, and inflammatory conditions [137]. Similarly in the Kwazulu-Natal region of South Africa, the stem bark decoction of *P. africana* is used to treat intercostal pain [138], a claim that was later confirmed by Stark et al. [82, 121]. In a parallel ethnobotanical investigation. Bii et al. [23] reported a potent in vitro pharmacological activity of methanolic extract derived from *P. africana* against *Streptococcus pneumoniae*, underscoring its efficacy and potential clinical relevance.

The potential effectiveness of *P. africana* in alleviating chest pain is attributed to its potent anti-inflammatory properties, which are inherent in the plant's phytochemicals; however, we must note that definitive scientific studies on this are lacking. Considerable antinociceptive and anti-inflammatory activities have been observed in the tannins of *P. africana* [73]. Likewise, due to their potent anti-inflammatory and analgesic activities, saponin compounds present may contribute to the analgesic effects of *P. africana* [139]. Borgi et al. [140] underscored the significant analgesic potential of saponins, noting their ability to suppress paw edoema, algesia, and nitrite production while maintaining cell viability. *In vivo* studies employing model animals have demonstrated that alkaloids from *P. africana* possess potent analgesic properties [141]. Consequently, the diverse classes of phytochemicals within *P. africana* might partially validate its efficacy in alleviating chest pain. While these findings suggest a plausible mechanism for *P*. *africana*'s efficacy in alleviating chest pain, it is imperative to conduct additional empirical studies to validate these claims conclusively.

### 3.12. Wound-Healing Effects

The historical application of medicinal plants to facilitate wound healing and prevent infections without significant adverse effects is well-documented [142, 143]. Notably, *P. africana* is frequently employed in the treatment of wounds within various African communities. Simbo [144] conducted an ethnobotanical study in Babungo, Northwest Region, Cameroon, and observed that *P. africana* is ethnomedicinally used to treat wounds and burns. This could potentially be due to the presence of secondary metabolites, such as tannins, alkaloids, flavonoids, and saponins, which confer wound-healing properties [124].

The substantial concentration of tannins in medicinal plants characterises their wound-healing and anti-inflammatory effects [142]. In addition, physiological effects such as accelerated blood coagulation, reduced blood pressure, and modulation of immune responses by tannins contribute to the wound-healing properties of this plant [33, 83]. Furthermore, the astringent properties of tannins in *P. africana* are known to expedite wound healing [128]. Tannins' antibacterial activity and their ability to enhance NIH3 T3 cell proliferation have been noted to promote wound shrinkage, improve healing rates, and facilitate the recovery of infectious wounds [125, 126]. Moreover, tannins have been shown to reduce *Staphylococcus aureus* colonization in wounds, contributing to improved quality of healing [130, 135, 145].

Alkaloids, recognized for their potent wound-healing activities, have demonstrated efficacy in dermal healing when topically applied [141, 146–148]. Flavonoids, as antioxidants, actively scavenge free radicals, preventing oxidative damage, and exhibit remarkable anti-inflammatory activities [149]. Their astringent nature, along with antimicrobial attributes, boosts the rates of epithelialization and wound contraction, thus promoting the healing process [33]. Moreover, previous reports show that flavonoids enhance collagen synthesis, by facilitating cross-linking in collagen fibres, shortening inflammation periods, and bestowing resistance to infections [150]. These are all critical components for amplifying the wound-healing continuum. Notably, Geethalakshmi et al. [151] reported that flavonoids, indeed, exhibit a wound-healing potential surpassing that of silver sulfadiazine—a reference drug for burn infection treatment.

Elsewhere, research demonstrated that saponins actively contribute to the treatment and management of various diseases, notably wound healing [140, 152]. Their unique ability to precipitate and aggregate red blood cells serves as a crucial factor in wounds' therapeutic process, by effectively halting bleeding [152, 153]. Moreover, studies indicate that saponins promote enhanced wound healing by stimulating wound contraction and facilitating increased collagen deposition [151, 154]. The persistent utilization of *P. africana* in traditional medicinal practices for wound healing finds its rationale in the pharmacological effects it possesses on promoting wound closure, largely due to the presence of specific phytochemicals. Nevertheless, extensive studies to unearth the full potential of this plant in wound healing, identification and characterization of the specific responsible compounds, and their action mechanisms may provide important insights into this plant's pharmacological potential and safety. This will, in turn, aid to translate the reported results into clinical applications upon thorough validation.

### 3.13. Safety and Toxicity

While traditional medicinal plants are commonly perceived as safe, it is imperative to recognize the potential for toxicity, necessitating caution [155, 156]. Numerous scientific investigations consistently affirm the nontoxic characteristics of *P. africana* bark extract in humans, even at higher doses [157]. A previous study by Karani et al. [158] noted that *P. africana* bark extract did not cause adverse when administered at a dose of 1000 mg/kg body weight to BALB/c mice. Elsewhere, repeated daily doses of aqueous extract of *P. africana* bark (1000 mg/kg body weight), administered orally for eight weeks, caused mild toxicity in rats [159, 160].

Conversely, an in vivo experiment, conducted over 4 weeks with a dosage of 300 mg/kg/day, demonstrated no observable effects as reviewed previously [73]. Although the probity analysis method calculated the lethal dose (LD_50_) for *P. africana* as up to 2201.207 mg/kg body weight [158]. A recent study proposed a relatively higher LD_50_ exceeding 5000 mg/kg body weight for *P. africana* extracts, denoting its safety [161]. Despite these complexities, the collective evidence underscores the overall nontoxic nature of the *P. africana* bark extract, providing a foundation for its continued exploration as a therapeutic agent. Nevertheless, cautious optimism and rigorous scrutiny remain paramount in leveraging traditional medicinal plants for therapeutic purposes, ensuring both efficacy and safety in clinical applications.

## 4. Limitations

This review has certain limitations. Firstly, the exclusion of unpublished information in the review process may introduce publication bias. Secondly, there is a possibility of overlooking studies or crucial information published on platforms beyond the scope of our focus. Nonetheless, this review significantly underscores the ethnopharmacological potential of *P. africana* in treating various ailments.

## 5. Conclusions and Future Directions

This comprehensive review offers valuable insights into the utilization of *Prunus africana*, highlighting its significance as a potent and versatile medicinal plant deeply rooted in traditional medicine. The plant's effectiveness extends across a wide range of medical conditions, both locally and worldwide, including benign prostatic hyperplasia, prostate cancer, chest discomforts, diabetes, wound management, malaria, gastrointestinal problems, and skin infections.

Despite numerous scientific studies documenting the phytochemical composition and therapeutic applications of *P. africana* stem bark, equivalent investigations for its leaves and roots are lacking, although their use is documented in traditional medicine. Hence, we recommend future research to undertake comparative analyses of the phytochemical composition and medicinal potential of the plant's stem bark, roots, and leaves. Furthermore, additional preclinical and clinical studies are imperative to validate the efficacy and safety of these *P. africana* phytochemicals, whether used individually or in combination, for potential integration into drug research and development.

Despite the plant's considerable therapeutic potential, unsustainable practices such as improper stem bark harvesting and illegal logging have rendered *P. africana*, a vulnerable species. Urgent efforts are essential to ensure its sustainable utilization and conservation throughout its distribution range.

## Figures and Tables

**Table 1:** Summary of ethnomedicinal uses of *P. africana*.

Part used	Condition	Preparation method	Administration route	References
Stem bark	Acquired immunodeficiency syndrome (AIDS)	Decoction	Oral	[20]
	Benign prostatic hyperplasia	Decoction/extracts	Oral	[16, 21, 22]
	Chest and intercostal pain, and respiratory conditions	Decoction	Oral	[14, 19, 23, 24]
	Diabetes	Decoction	Oral	[25]
	Erectile dysfunction		Oral	[16]
	Fever	Decoction	Oral	[14, 16, 23, 25]
	Gastrointestinal conditions	Decoction/infusion	Oral	[14, 19, 20, 26, 27]
	Gonorrhea	Decoction	Oral	[14, 21]
	Hypertension	Decoction	Oral	[28–30]
	Kidney diseases	Decoction	Oral	[16, 23]
	Malaria	Decoction	Oral	[19, 20, 23, 24]
	Mental illness	Decoction	Oral	[24]
	Prostate cancer	Decoction	Oral	[19, 20, 31, 32]
	Purgative	Decoction	Oral	[14, 20]
	Skin infections	Powder	Topical	[19]
	Typhoid	Decoction	Oral	[19]
	Ulcers	Decoction	Oral	[19]
	Urinary disorders	Decoction	Oral	[16]
	Wound healing	Powder	Topical	[33]
	Inflammation	Decoction	Oral	[16]

Roots	Chest (intercostal) pain	Decoction	Oral	[19, 34]
	Gastrointestinal conditions	Decoction	Oral	[34]
	Enlarged prostate	Decoction/infusion	Oral	[28]
	Urinary disorders	Decoction	Oral	[20]

Leaves	Gastrointestinal conditions	Decoction	Oral	[14]
	Appetite	Infusion	Oral	[20]
	Fever	Inhalant	Inhaled/oral	[20]
	Insanity	Decoction	Oral	[14]

Fruits	Gastrointestinal conditions	Decoction	Oral	[14, 35]
	Chest and intercostal pain	Decoction	Oral	[14, 35]

**Table 2:** Summary of *P. africana* phytochemical**s**.

Phytochemical compound	Compound structure	References
Atraric acid	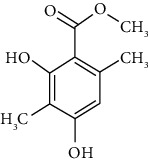	[16, 39, 40]

Ferulic acid	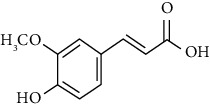	[16, 39, 40]

Lauric acid	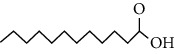	[16, 39, 40]

Myristic acid	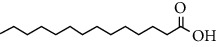	[16, 39, 40]

N-butylbenzene-sulfonamide	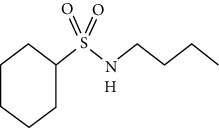	[16, 39]

Oleanolic acid	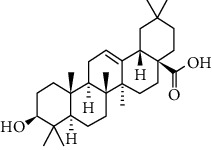	[16, 39, 40]

Ursolic acid	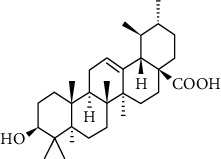	[16, 39–41]

*β*-amyrin	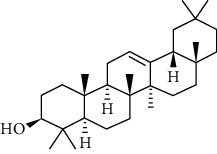	[16, 39–41]

*β*-sitosterol	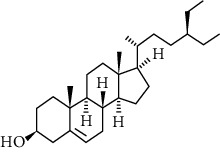	[16, 39–41]

*β*-sitostenone	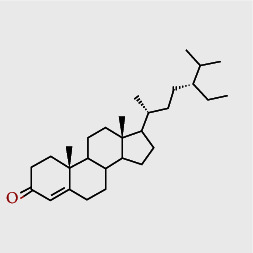	[41]

**Table 3:** Summary of biological/pharmacological activities of notable *P. africana* phytochemicals.

Phytochemical	Pharmacological effects	References
Ursolic acid	(i) *In vivo* inhibition of acetylcholinesterase activity in scopolamine-induced cognitive-impaired Swiss albino mice to improve cognition and performance in a passive avoidance task	[41]

Quercetin and quercetin 3,30-dimethyl ether-40-glucoside	(i) *In vitro* downregulation of Bcl-2 resulting in PC-3 cells' apoptosis	[39]
	(ii) *In vivo* inhibition of alpha-reductase enzyme activity to inhibit prostate inflammation	[16]
	(iii) Activates signalling pathways that regulate synaptic plasticity and long-term potentiation, ensuring neuronal integrity	[41, 49]
	(iv) Ameliorate neuroinflammation in AD	

*β*-sitostenone	(i) *In vivo* anti-inflammatory, neuroprotective, and antioxidant properties	[41, 50]
	(ii) *In vivo* reduction of AChE, TNF-*α*, and corticosterone activities, and improves antioxidant enzymes' activity	

Chlorogenic acid	(i) *In vivo* inhibition of AChE activity in the frontal cortex and hippocampus based on *ex vivo* studies	[41, 51]

Apigenin	(i) *In vivo* anti-Alzheimer's, antidiabetic, antioxidant, and anti-inflammatory properties	[41, 52]

Catechin	(i) *In vitro* antioxidant activity	[53, 54]

Oleanolic acid	(i) Targets the AMPK in PC-3 cells *in vitro* to inhibit key metabolic pathways leading to apoptosis	[55, 56]
	(ii) Inhibits IFN-*γ*, nitric oxide synthase, and cyclooxygenase-2 in rat macrophages	[57]
	(iii) Induces phase-2 response by increasing heme oxygenase-1 and NADH-quinone oxidoreductase, to prevent cells' damage from free radicals and electrophiles	[41, 57]

*β*-amyrin	(i) Exhibits *in vitro* cytotoxicity to PC-3 cells	[39, 58]

Atraric acid	(i) Possess antiandrogenic activity with antiproliferative effects against PC-3 cells	[39, 59]

Ferulic acid	(i) Promotes *in vitro* PC-3 cells' apoptosis	[39, 58]
	(ii) *In vivo* inhibition of the alpha-reductase enzyme activity to reduce prostate inflammation	[16, 25, 59]

N-butylbenzene-sulfonamide	(i) Promotes apoptosis of PC-3 cells *in vitro*	[39, 59]

*β*-sitosterol	(i) Exhibits *in vitro* cytotoxicity and apoptotic effects against the PC-3 and LNCaP cells	[39, 59]
	(ii) Inhibits alpha-reductase enzyme activity, reducing prostate inflammation	[16, 37, 39]

Lauric acid	(i) *In vitro* inhibition of 5-*α*-reductase enzyme to prevent testosterone conversion to dihydrotestosterone	[39, 60]

Myristic acid	(i) *In vitro* antioxidant activity	[41, 42]

Flavonols	(i) *In vitro* antiproliferative activity against prostate cancer cells	[47]

Terpenoids	(i) Antimalarial activity by arresting *P. falciparum* growth and by inhibiting its biosynthesis of isoprenoids	[12, 61]

Tannins	(i) *In vitro* antimicrobial and antiparasitic activities to avert gastrointestinal diseases	[46]

Tannins	(i) *In vitro* antimicrobial activities against bacterial and fungal strains associated with skin infections	[23, 46, 61–64]

Tannins	(i) *In vitro* inhibition of *Streptococcus pneumonia* growth to alleviate respiratory disease	[23, 46, 61–64]

Tannins	(i) Possess *in vitro* antimicrobial and wound healing activities	[23, 46, 61–64]

## Data Availability

All data are presented within the manuscript; however, any additional information may be provided by the authors upon reasonable request.

## References

[1] James P. B., Wardle J., Steel A., Adams J. (2018). Traditional, complementary and alternative medicine use in Sub-Saharan Africa: a systematic review. *BMJ Global Health*.

[2] Che C. T., George V., Ijinu T. P., Pushpangadan P., Andrae-Marobela K. (2017). Traditional medicine. *Pharmacognosy: Fundamentals, Applications and Strategy*.

[3] WHO (2013). *Traditional Medicine: A Report by the Secretariat*.

[4] Salmerón-Manzano E., Garrido-Cardenas J. A., Manzano-Agugliaro F. (2020). Worldwide research trends on medicinal plants. *International Journal of Environmental Research and Public Health*.

[5] van Wyk A. S., Prinsloo G. (2018). Medicinal plant harvesting, sustainability and cultivation in South Africa. *Biological Conservation*.

[6] Cunningham A. B., Mbenkum F. T. (1993). *Sustainability of Harvesting Prunus Africana Bark in Cameroon A Medicinal Plant in International Trade*.

[7] de Lacey S., Smith C. (2011). Traditional Chinese medicine. *How to Improve Your ART Success Rates: An Evidence-Based Review of Adjuncts to IVF*.

[8] Picking D., Delgoda R., Vandebroek I. (2019). Traditional knowledge systems and the role of traditional medicine in Jamaica, CAB reviews: perspectives in agriculture, veterinary science. *Nutrition and Natural Resources*.

[9] WHO (2021). *Global Antimicrobial Resistance and Use Surveillance System (GLASS) Report*.

[10] European Antimicrobial Resistance Collaborators (2022). The burden of bacterial antimicrobial resistance in the WHO European region in 2019: a cross-country systematic analysis. *The Lancet Public Health*.

[11] Wang H., Abbas K. M., Abbasifard M. (2020). Global age-sex-specific fertility, mortality, healthy life expectancy (HALE), and population estimates in 204 countries and territories, 1950–2019: a comprehensive demographic analysis for the Global Burden of Disease Study 2019. *The Lancet*.

[12] Rubegeta E., Makolo F., Kamatou G. (2023). The African cherry: a review of the botany, traditional uses, phytochemistry, and biological activities of Prunus africana (Hook.f.) Kalkman. *Journal of Ethnopharmacology*.

[13] Cunningham A. B. (1993). African medicinal plants, setting priorities at the interface between conseivation and primary healthcare. *People and Plants Workmg Paper*.

[14] Stewart K. M. (2003). The african cherry (prunus africana): from Hoe-Handles to the international herb market. *Economic Botany*.

[15] Fourneau C., Hocquemiller R., Cav A. (1996). Triterpenes from prunus africana bark. *Phytochemistry*.

[16] Wavinya Nyamai D., Wambua F., Matheri F. (2015). Phytochemical profile of prunus africana stem bark from Kenya Pharmacognosy and natural products phytochemical profile of prunus africana stem bark from Kenya. *Journal of Pharmacognosy and Natural Products*.

[17] Higgins J. P. T., Altman D. G., Gotzsche P. C. (2011). The Cochrane Collaboration’s tool for assessing risk of bias in randomised trials. *BMJ (Online)*.

[18] Moher D., Shamseer L., Clarke M. (2015). Preferred reporting items for systematic review and meta-analysis protocols (PRISMA-P) 2015 statement. *Systematic Reviews*.

[19] Koros H. (2016). Uses and conservation of prunus africana (Hook. F.) kalkman in Nandi forests article in Wuhan University. *Journal of Natural Sciences*.

[20] Ochwang’i D. O., Kimwele C. N., Oduma J. A., Gathumbi P. K., Mbaria J. M., Kiama S. G. (2014). Medicinal plants used in treatment and management of cancer in Kakamega County, Kenya, Kenya. *Journal of Ethnopharmacology*.

[21] Njoroge G. N., Bussmann R. W. (2006). Traditional management of ear, nose and throat (ENT) diseases in Central Kenya. *Journal of Ethnobiology and Ethnomedicine*.

[22] Kareru P. G., Kenji G. M., Mungai G., Keriko J. M., Mungai G., Keriko J. M. (2006). Traditional medicines among the Embu and Mbeere peoples of Kenya. *African Journal of Traditional, Complementary and Alternative Medicines: AJTCAM*.

[23] Bii C., Korir K. R., Rugutt J., Mutai C. (2010). The potential use of Prunus africana for the control, treatment and management of common fungal and bacterial infections. *Journal of Medicinal Plants Research*.

[24] Jiofack T., Ayissi I., Fokunang C., Guedje N., Kemeuze V. (2009). Ethnobotany and phytomedicine of the upper Nyong valley forest in Cameroon. *African Journal of Pharmacy and Pharmacology*.

[25] Godfrey Mutuma G., Joseph N., King’ori M. A., Silas K. (2020). Phytochemical and anti-inflammatory analysis of prunus africana bark extract. *Research Journal of Pharmacognosy*.

[26] Kisangau D. P., Kauti M., Mwobobia R., Kanui T., Musimba N., License C. A. (2017). Traditional knowledge on Use of medicinal plants in Kitui County, Kenya. *International Journal of Ethnobiology& Ethnomedicine*.

[27] Kisangau D. P., Herrmann T. M. (2007). Utilization and conservation of medicinal plants used for primary health care in Makueni district, Kenya. *International Journal of Biodiversity Science and Management*.

[28] Kipkore W., Wanjohi B., Rono H., Kigen G. (2014). A study of the medicinal plants used by the Marakwet Community in Kenya. *Journal of Ethnobiology and Ethnomedicine*.

[29] Kigen G., Kamuren Z., Njiru E., Wanjohi B., Kipkore W. (2019). Ethnomedical survey of the plants used by traditional healers in Narok County, Kenya. *Evidence-based Complementary and Alternative Medicine*.

[30] Kigen G. K., Ronoh H. K., Kipkore W. K., Rotich J. K. (2013). Current trends of traditional herbal medicine practice in Kenya: a review. *Journal of Pharmacology and Therapeutics*.

[31] Tugume P., Kakudidi E. K., Buyinza M. (2016). Ethnobotanical survey of medicinal plant species used by communities around Mabira Central Forest Reserve. *Uganda, J Ethnobiol Ethnomed*.

[32] Tugume P., Nyakoojo C. (2019). Ethno-pharmacological survey of herbal remedies used in the treatment of paediatric diseases in Buhunga parish, Rukungiri District, Uganda. *BMC Complementary and Alternative Medicine*.

[33] Hanbisa S., Tadesse W. T., Abula T. (2023). Evaluation of wound healing activity of 80% Methanol stem-bark extract and Solvent fractions of prunus africana (Hook.f.) kalkman (Rosaceae) in mice. *Journal of Experimental Pharmacology*.

[34] Bodeker G., Van’T Klooster C., Weisbord E. (2014). Prunus africana (Hook.f.) Kalkman: the overexploitation of a medicinal plant species and its legal context. *Journal of Alternative and Complementary Medicine*.

[35] Josephine Ozioma E.-O., Antoinette Nwamaka Chinwe O. (2019). Herbal medicines in african traditional medicine, herbal medicine. *Herbal Medicine*.

[36] Stewart K. M. (2003). The African cherry (Prunus africana): can lessons be learned from an over-exploited medicinal tree?. *Journal of Ethnopharmacology*.

[37] Jepkorir M., Nyanjom S. G., Kamau S., Chepng’etich J., Kipkoech G., Mwitari P. G. (2023). In vivo anti-inflammatory activity, safety and gene expression profiles of Carissa edulis, Withania somnifera, Prunus africana and Rhamnus prinoides for potential management of rheumatoid arthritis. *Scientific African*.

[38] Onyancha J. M., Moriasi G. A., Nyandoro V. O., Onyancha B. M., Onsinyo J. M. (2023). Ethnomedicinal review of plants utilized by the Abagusii people of Western Kenya. *Advances in Traditional Medicine*.

[39] Komakech R., Kang Y., Lee J. H., Omujal F. (2017). A review of the potential of phytochemicals from prunus Africana (Hook f.) kalkman stem bark for chemoprevention and chemotherapy of prostate cancer. *Evidence-based Complementary and Alternative Medicine*.

[40] Kadu C. A. C., Parich A., Schueler S. (2012). Bioactive constituents in Prunus africana: Geographical variation throughout Africa and associations with environmental and genetic parameters. *Phytochemistry*.

[41] Ngai D. N., Kibiti C. M., Ngugi M. P. (2022). Cognitive enhancing effects and anticholinesterase activity of stem bark and leaf extracts of Prunus africana. *Heliyon*.

[42] Hass M. A., Nowak D. M., Leonova E., Levin R. M., Longhurst P. A. (1999). Identification of components of Prunus africana extract that inhibit lipid peroxidation. *Phytomedicine*.

[43] Begeno T. A., Teka A. E., Bafa T. A., Nassir W. B. (2020). Phytochemical investigation and characterization on the leaf extract of prunus africana. *International Research Journal of Pure and Applied Chemistry*.

[44] Nambooze J., Erukainure O. L., Chukwuma C. I. (2022). Phytochemistry of Prunus africana and its therapeutic effect against prostate cancer. *Comparative Clinical Pathology*.

[45] Teshale A. B. (2020). Phytochemical investigation and characterization on the root bark extract of prunus africana. *Chemistry and Materials Research*.

[46] Ngule C., Francis R. (2016). Chemical constituents screening and in vitro antibacterial assessment of prunus africana bark hydromethanolic extract. https://www.researchgate.net/publication/288993461.

[47] Letoyah A. Y., Antiproliferative Activity (2017). *Phytochemical Composition and Toxicity Studies of Fagaropsis Angolensis and Prunus Africana Crude Extracts*.

[48] Moriasi G., Ireri A., Ngugi M. P. (2020). In vitro antioxidant activities of the aqueous and methanolic stem bark extracts of Piliostigma thonningii (Schum.). *Journal of Evidence-Based Integrative Medicine*.

[49] Olthof M. R., Hollman P. C. H., Buijsman M. N. C. P., Van Amelsvoort J. M. M., Katan M. B. (2003). Human Nutrition and Metabolism Chlorogenic Acid, Quercetin-3-Rutinoside and Black Tea Phenols Are Extensively Metabolized in Humans. *The Journal of Nutrition*.

[50] Âbraham I. M., Harkany T., Horvath K. M., Luiten P. G. M. (2001). Action of Glucocorticoids on survival of Nerve cells: promoting neurodegeneration or neuroprotection?. *Journal of Neuroendocrinology*.

[51] Orhan I., Şener B., Choudhary M. I., Khalid A. (2004). Acetylcholinesterase and butyrylcholinesterase inhibitory activity of some Turkish medicinal plants. *Journal of Ethnopharmacology*.

[52] Colizzi C. (2019). The protective effects of polyphenols on Alzheimer’s disease: a systematic review. *Alzheimer’s and Dementia: Translational Research and Clinical Interventions*.

[53] Zanwar A. A., Badole S. L., Shende P. S., Hegde M. V., Bodhankar S. L. (2013). Antioxidant role of catechin in health and disease. *Polyphenols in Human Health and Disease*.

[54] Koch W., Kukula-Koch W., Głowniak K. (2017). Catechin composition and antioxidant activity of black teas in relation to brewing time. *Journal of AOAC International*.

[55] Liang J., Zhou Y., Cheng X. (2023). Baicalin Attenuates H2O2-induced oxidative stress by regulating the AMPK/Nrf2 Signaling pathway in IPEC-J2 cells. *International Journal of Molecular Sciences*.

[56] Gejjalagere Honnappa C., Mazhuvancherry Kesavan U. (2016). A concise review on advances in development of small molecule anti-inflammatory therapeutics emphasising AMPK: an emerging target. *International Journal of Immunopathology and Pharmacology*.

[57] Yang Y., Zhang Z., Li S., Ye X., Li X., He K. (2014). Synergy effects of herb extracts: pharmacokinetics and pharmacodynamic basis. *Fitoterapia*.

[58] Asuzu P. C., Aryee A. N. A., Trompeter N. (1990). Vitro Assessment of Efficacy and Cytotoxicity of Prunus Africana Extracts on Prostate Cancer C4-2 Cells 2 3 Department of Biomedical Engineering. https://www.biorxiv.org/content/10.1101/2021.03.14.435338v1.full.

[59] Komakech R., Yim N. H., Shim K. S. (2022). Root extract of a Micropropagated prunus africana medicinal plant induced apoptosis in human prostate cancer cells (PC-3) via Caspase-3 activation. *Evidence-based Complementary and Alternative Medicine*.

[60] Krzywik J., Mozga W., Aminpour M. (2020). Synthesis, antiproliferative activity and molecular docking studies of novel doubly modified colchicine amides and sulfonamides as anticancer agents. *Molecules*.

[61] Bettarini F., Borgonovi G. E., Fiorani T. (1993). Antiparasitic compounds from East African plants: Isolation and biological activity of anonaine, matricarianol, canthin-6-one and caryophyllene oxide. *International Journal of Tropical Insect Science*.

[62] Mwitari P. G., Ayeka P. A., Ondicho J., Matu E. N., Bii C. C. (2013). Antimicrobial activity and probable mechanisms of action of medicinal plants of Kenya: Withania somnifera, Warbugia ugandensis, prunus africana and Plectrunthus barbatus. *PLoS One*.

[63] Baloyi I. T., Cosa S., Combrinck S., Leonard C. M., Viljoen A. M. (2019). Anti-quorum sensing and antimicrobial activities of South African medicinal plants against uropathogens. *South African Journal of Botany*.

[64] Das S. (2017). Antibacterial activity OF PRUNUS africana stem bark extract against SHIGELLA SPP. *World Journal of Pharmacy and Pharmaceutical Sciences*.

[65] Rastogi S., Pandey M. M., Rawat A. K. S. (2016). Traditional herbs: a remedy for cardiovascular disorders. *Phytomedicine*.

[66] Haidan Yuan G. P., Ma Q., Ye L. (2016). The traditional medicine and modern medicine from natural products. *Molecules*.

[67] Othman C. N., Farooqui M. (2015). Traditional and complementary medicine. *Procedia—Social and Behavioral Sciences*.

[68] Bosman A., Mendis K. N. (2007). A major Transition in malaria treatment: the Adoption and Deployment of Artemisinin-based combination therapies. *The American Journal of Tropical Medicine and Hygiene*.

[69] Hacioglu M., Dosler S., Birteksoz Tan A. S., Otuk G. (2017). Antimicrobial activities of widely consumed herbal teas, alone or in combination with antibiotics: an in vitro study. *PeerJ*.

[70] Sarian M. N., Ahmed Q. U., Mat So’Ad S. Z. (2017). Antioxidant and antidiabetic effects of flavonoids: a structure-activity relationship based study. *BioMed Research International*.

[71] Mordeniz C. (2019). Integration of traditional and complementary medicine into evidence-based clinical practice. *Traditional and Complementary Medicine*.

[72] Sen S., Chakraborty R. (2017). Revival, modernization and integration of Indian traditional herbal medicine in clinical practice: importance, challenges and future. *Journal of Traditional and Complementary Medicine*.

[73] Komakech R., Kang Y. (2019). Ethnopharmacological potential of African cherry Prunus africana. *Journal of Herbal Medicine*.

[74] Jena A. K., Vasisht K., Sharma N., Kaur R., Dhingra M. S., Karan M. (2016). Amelioration of testosterone induced benign prostatic hyperplasia by Prunus species. *Journal of Ethnopharmacology*.

[75] Nyamai D. W., Burugu M. W., Ng’ang’a M. M., Muchugi A. N. (2022). The phytochemical profiles and growth of Prunus africana in Kenya. *Asian Journal of Natural Product Biochemistry*.

[76] Eisermann K., Fraizer G. (2017). The androgen receptor and VEGF: mechanisms of androgen-regulated angiogenesis in prostate cancer. *Cancers*.

[77] Zi H., He S. H., Leng X. Y. (2021). Global, regional, and national burden of kidney, bladder, and prostate cancers and their attributable risk factors, 1990–2019. *Military Medical Research*.

[78] Desai A. G., Qazi G. N., Ganju R. K. (2008). Medicinal plants and cancer chemoprevention. *Current Drug Metabolism*.

[79] Siddiqui A. J., Jahan S., Singh R. (2022). Plants in anticancer drug discovery: from molecular mechanism to chemoprevention. *BioMed Research International*.

[80] Thompson R. Q., Katz D., Sheehan B. (2019). Chemical comparison of Prunus africana bark and pygeum products marketed for prostate health. *Journal of Pharmaceutical and Biomedical Analysis*.

[81] Shrihastini V., Muthuramalingam P., Adarshan S. (2021). Plant derived bioactive compounds, their anti‐cancer effects and in silico approaches as an alternative target treatment strategy for breast cancer: an updated overview. *Cancers*.

[82] Kuete V., Viertel K., Efferth T. (2016). Antiproliferative potential of african medicinal plants. *Medicinal Plant Research in Africa: Pharmacology and Chemistry, Saint Louis, US*.

[83] Chung K. T., Wong T. Y., Wei C. I., Huang Y. W., Lin Y. (1998). Tannins and human health: a review. *Critical Reviews in Food Science and Nutrition*.

[84] Yiaile A. L., Mbaria J. M., Ole-Mapenay I. M., Okumu M. O., Hadun A. H., Onyancha J. M. (2018). Preliminary screening of Crude extracts of Fagaropsis Angolensis for anticancer activity. *Pharmacognosy Communications*.

[85] Moriasi G., Kibiti C., Ngugi M. (2021). In vivo antidiabetic efficacy, Mineral Element composition, and Qualitative phytochemistry of the aqueous leaf extracts of Pentas zanzibarica (Klotzsch.) Vatke and Olea europaea subspecies africana (Mill.). *Journal of Advanced Biotechnology and Experimental Therapeutics*.

[86] Moriasi G., Ireri A., Ngugi M. (2020). Cognitive-enhancing, Ex vivo Antilipid peroxidation and Qualitative phytochemical evaluation of the aqueous and methanolic stem bark extracts of Lonchocarpus eriocalyx (Harms.). *Biochemistry Research International*.

[87] Muruthi C. W., Ngugi M. P., Runo S. M., Mwitari P. G. (2023). In vitro antiproliferative effects and phytochemical characterization of Carissa edulis ((Forssk) Vahl) and Pappea capensis (Eckyl and Zeyh) extracts. *Journal of Evidence-Based Integrative Medicine*.

[88] International Diabetes Federation (2019). *IDF Diabetes Atlas*.

[89] WHO (2016). Global Report on Diabetes. http://www.who.int/about/licensing/copyright_form/index.html%0A.

[90] Ullah H., De Filippis A., Khan H., Xiao J., Daglia M. (2020). An overview of the health benefits of Prunus species with special reference to metabolic syndrome risk factors. *Food and Chemical Toxicology*.

[91] Barbagallo M. (2015). Magnesium and type 2 diabetes. *World Journal of Diabetes*.

[92] Obafemi T. O., Akinmoladun A. C., Olaleye M. T., Agboade S. O., Onasanya A. A. (2017). Antidiabetic potential of methanolic and flavonoid-rich leaf extracts of Synsepalum dulcificum in type 2 diabetic rats. *Journal of Ayurveda and Integrative Medicine*.

[93] Andrade-Cetto A., Cruz E. C., Cabello-Hernández C. A., Cárdenas-Vázquez R. (2019). Hypoglycemic activity of medicinal plants used among the Cakchiquels in Guatemala for the treatment of type 2 diabetes. *Evidence-based Complementary and Alternative Medicine*.

[94] Wang D., Li Y., Hou G. (2010). Pygeum africanum: effect on oxidative stress in early diabetes-induced bladder. *International Urology and Nephrology*.

[95] Maina J. K., Kareru P. G., Gatebe E. G., Githira P. N., Mutembei J. K. (2014). Hypoglycemic Effects of Selected Herbal Drug Formulations from the Kenyan Market. http://scholarsresearchlibrary.com/archive.html.

[96] Singh R., Arif T., Khan I., Sharma P. (2014). Phytochemicals in antidiabetic drug discovery. *Journal of Biomedical and Therapeutic Sciences*.

[97] Ben Younes A., Ben Salem M., El Abed H., Jarraya R. (2018). Phytochemical screening and antidiabetic, antihyperlipidemic, and antioxidant properties of anthyllis henoniana (COSS.) flowers extracts in an alloxan-induced rats model of diabetes. *Evidence-based Complementary and Alternative Medicine*.

[98] Tafesse T. B., Hymete A., Mekonnen Y., Tadesse M. (2017). Antidiabetic activity and phytochemical screening of extracts of the leaves of Ajuga remota Benth on alloxan-induced diabetic mice. *BMC Complementary and Alternative Medicine*.

[99] Vinayagam R., Jayachandran M., Xu B. (2016). Antidiabetic effects of Simple Phenolic acids: a comprehensive review. *Phytotherapy Research*.

[100] Yongchaiyudha S., Rungpitarangsi V., Bunyapraphatsara N., Chokechaijaroenporn O. (1996). Antidiabetic activity of Aloe vera L. juice. I. Clinical trial in new cases of diabetes mellitus. *Phytomedicine*.

[101] Tiong S. H., Looi C. Y., Hazni H. (2013). Antidiabetic and antioxidant properties of alkaloids from Catharanthus roseus (L.) G. Don. *Molecules*.

[102] Mutembei J. K., Kareru P. G., Madivoli E. S. (2018). Phytochemical and antimicrobial evaluation of selected medicinal plants in Meru community of Kenya. *Journal of Medicinal Plants for Economic Development*.

[103] Gakuubi M. M., Wanzala W. (2012). A survey of plants and plant products traditionally used in livestock health management in Buuri district, Meru County, Kenya. *Journal of Ethnobiology and Ethnomedicine*.

[104] Muthaura C. N., Keriko J. M., Mutai C. (2015). Antiplasmodial potential of traditional antimalarial phytotherapy remedies used by the Kwale community of the Kenyan Coast. *Journal of Ethnopharmacology*.

[105] Muthaura C. N., Rukunga G. M., Chhabra S. C. (2007). Antimalarial activity of some plants traditionally used in treatment of malaria in Kwale district of Kenya. *Journal of Ethnopharmacology*.

[106] Gathirwa J. W., Rukunga G. M., Mwitari P. G. (2011). Traditional herbal antimalarial therapy in Kilifi district, Kenya. *Journal of Ethnopharmacology*.

[107] Onguéné P. A., Ntie-Kang F., Mbah J. A. (2014). The potential of anti-malarial compounds derived from African medicinal plants, part III: an in silico evaluation of drug metabolism and pharmacokinetics profiling. *Organic and Medicinal Chemistry Letters*.

[108] Lutgen P. (2018). Tannins in Artemisia: the hidden treasure of prophylaxis. *Pharmacy and Pharmacology International Journal*.

[109] Serge K. B., Serges O. A., Ludovic M. (2015). Vitro behaviour of plasmodium falciparum strains by alkaloids and tannins extracted from root of mitragyna inermis, a medicinal plant. *International Journal of Current Pharmaceutical Research*.

[110] Lehane A. M., Saliba K. J. (2008). Common dietary flavonoids inhibit the growth of the intraerythrocytic malaria parasite. *BMC Research Notes*.

[111] Rodrigues Goulart H., Kimura E. A., Peres V. J., Couto A. S., Aquino Duarte F. A., Katzin A. M. (2004). Terpenes arrest parasite development and inhibit biosynthesis of isoprenoids in Plasmodium falciparum. *Antimicrobial Agents and Chemotherapy*.

[112] Guggisberg A. M., Amthor R. E., Odom A. R. (2014). Isoprenoid biosynthesis in Plasmodium falciparum. *Eukaryotic Cell*.

[113] Murata T., Miyase T., Muregi F. W. (2008). Antiplasmodial triterpenoids from Ekebergia capensis. *Journal of Natural Products*.

[114] Waiganjo B., Moriasi G., Onyancha J., Elias N., Muregi F. (2020). Antiplasmodial and Cytotoxic activities of extracts of selected medicinal plants used to treat malaria in Embu County, Kenya. *Journal of Parasitology Research*.

[115] Carrasco J. M. D., Casanova N. A., Miyakawa M. E. F. (2019). Microbiota, gut health and chicken productivity: what is the connection?. *Microorganisms*.

[116] Śliżewska K., Markowiak P., Żbikowski A., Szeleszczuk P. (2019). Effects of synbiotics on the gut microbiota, blood and rearing parameters of chickens. *FEMS Microbiology Letters*.

[117] Hakansson A., Molin G. (2011). Gut microbiota and inflammation. *Nutrients*.

[118] Gagliardi A., Totino V., Cacciotti F. (2018). Rebuilding the gut microbiota ecosystem. *International Journal of Environmental Research and Public Health*.

[119] Fan Y., Pedersen O. (2021). Gut microbiota in human metabolic health and disease. *Nature Reviews Microbiology*.

[120] Honneffer J. B., Minamoto Y., Suchodolski J. S. (2014). Microbiota alterations in acute and chronic gastrointestinal inflammation of cats and dogs. *World Journal of Gastroenterology*.

[121] Stark T. D., Mtui D. J., Balemba O. B. (2013). Ethnopharmacological survey of plants used in the traditional treatment of gastrointestinal pain, inflammation and diarrhea in Africa: future perspectives for integration into modern medicine. *Animals*.

[122] Amri E., Kisangau D. P. (2012). Ethnomedicinal study of plants used in villages around Kimboza forest reserve in Morogoro, Tanzania. *Journal of Ethnobiology and Ethnomedicine*.

[123] Eldeen I. M. S., Elgorashi E. E., Van Staden J. (2005). Antibacterial, anti-inflammatory, anti-cholinesterase and mutagenic effects of extracts obtained from some trees used in South African traditional medicine. *Journal of Ethnopharmacology*.

[124] Chrispus Ngule M., Ndiku M. H., Ramesh F. (2014). Chemical Constituents Screening and in Vitro Antibacterial Assessment of Prunus Africana Bark Hydromethanolic Extract. https://www.iiste.org.

[125] Ky I., Le Floch A., Zeng L., Pechamat L., Jourdes M., Teissedre P. L. (2015). Tannins. *Encyclopedia of Food and Health*.

[126] Serrano J., Puupponen-Pimiä R., Dauer A., Aura A. M., Saura-Calixto F. (2009). Tannins: Current knowledge of food sources, intake, bioavailability and biological effects. *Molecular Nutrition and Food Research*.

[127] Scalbert A. (1991). Antimicrobial properties of tannins. *Phytochemistry*.

[128] Ashok P., Upadhyaya K. (2012). Tannins are astringent. *Journal of Pharmacognosy and Phytochemistry*.

[129] Buzzini P., Arapitsas P., Goretti M. (2008). Antimicrobial and antiviral activity of Hydrolysable tannins. *Mini-Reviews in Medicinal Chemistry*.

[130] Salih E. Y. A., Kanninen M., Sipi M. (2017). Tannins, flavonoids and stilbenes in extracts of African savanna woodland trees Terminalia brownii, Terminalia laxiflora and Anogeissus leiocarpus showing promising antibacterial potential. *South African Journal of Botany*.

[131] Kimathi P. K., Maitho T. E., Mbaria J. M., Moriasi G. A. (2022). Antidiarrheal, antimicrobial, and toxic effects of the aqueous and methanolic leaf and fruit extracts of Cucumis dipsaceus (Ehrenb. Ex Spach.). *Journal of HerbMed Pharmacology*.

[132] De Lira Mota K. S., Dias G. E. N., Pinto M. E. F. (2009). Flavonoids with gastroprotective activity. *Molecules*.

[133] Bor T., Aljaloud S. O., Gyawali R., Ibrahim S. A. (2016). Antimicrobials from Herbs, Spices, and Plants. *Herbs and Spices- New Advances*.

[134] Abreu A. C., McBain A. J., Simões M. (2012). Plants as sources of new antimicrobials and resistance-modifying agents. *Natural Product Reports*.

[135] Akiyama H., Fujii K., Yamasaki O., Oono T., Iwatsuki K. (2001). Antibacterial action of several tannins against Staphylococcus aureus. *Journal of Antimicrobial Chemotherapy*.

[136] Mabhiza D., Chitemerere T., Mukanganyama S. (2016). Antibacterial properties of alkaloid extracts from *Callistemon citrinus* and *Vernonia adoensis* against *Staphylococcus aureus* and *Pseudomonas aeruginosa*. *International Journal of Medicinal Chemistry*.

[137] Kuete V. (2014). *Toxicological Survey of African Medicinal Plants, First*.

[138] Grace O. M., Prendergast H. D. V., Jäger A. K., Van Staden J., van Wyk A. (2003). Bark medicines used in traditional healthcare in KwaZulu-Natal, South Africa: an inventory. *South African Journal of Botany*.

[139] Küpeli Akkol E., Irem Tatli I., Akdemir Z. S. (2007). Antinociceptive and anti-inflammatory effects of saponin and Iridoid Glycosides from Verbascum pterocalycinum var. mutense Hub. *Morphology*.

[140] Borgi W., Recio M. C., Ríos J. L., Chouchane N. (2008). Anti-inflammatory and analgesic activities of flavonoid and saponin fractions from Zizyphus lotus (L.) Lam. *South African Journal of Botany*.

[141] Kumar Sutradhar R., Chnadra Bachar S., Roy T. (2007). Anti-inflammatory and Analgesic Alkaloid from Sida Cordifolia Linn. https://www.researchgate.net/publication/346471900.

[142] Vatnikov Y., Shabunin S., Kulikov E. (2020). Effectiveness of biologically active substances from Hypericum Perforatum L. in the complex treatment of purulent wounds. *International Journal of Pharmaceutical Research*.

[143] Atef N. M., Shanab S. M., Negm S. I., Abbas Y. A. (2019). Evaluation of antimicrobial activity of some plant extracts against antibiotic susceptible and resistant bacterial strains causing wound infection. *Bulletin of the National Research Centre*.

[144] Simbo D. J. (2010). An ethnobotanical survey of medicinal plants in Babungo, Northwest Region, Cameroon. http://www.ethnobiomed.com/content/6/1/8.

[145] Ukoha P. O., Cemaluk E. A. C., Nnamdi O. L., Madus E. P. (2011). Tannins and other phytochemical of the Samanaea saman pods and their antimicrobial activities. *African Journal of Pure and Applied Chemistry*.

[146] Cushnie T. T., Cushnie B., Lamb A. J. (2014). Alkaloids: an overview of their antibacterial, antibiotic-enhancing and antivirulence activities. *International Journal of Antimicrobial Agents*.

[147] Debnath B., Singh W. S., Das M. (2018). Role of plant alkaloids on human health: a review of biological activities. *Materials Today Chemistry*.

[148] Formagio A. S. N., de Oliveira Junior P. C., Volobuff C. R. F. (2019). Anti-inflammatory activity of methanolic extract and an alkaloid from Palicourea crocea (Sw.) Roem and Schult. *Inflammation*.

[149] Akimat E. K., Omwenga G. I., Moriasi G. A., Ngugi M. P. (2021). Antioxidant, anti-inflammatory, acute oral toxicity, and Qualitative phytochemistry of the aqueous root extract of Launaea cornuta (Hochst. Ex Oliv. and hiern.). *Journal of Evidence-Based Integrative Medicine*.

[150] Altan A., Aras M. H., Damlar I., Gokce H., Ozcan O., Alpaslan C. (2019). The effect of Hypericum Perforatum on wound healing of oral mucosa in diabetic rats. *European Oral Research*.

[151] Geethalakshmi R., Sakravarthi C., Kritika T., Arul Kirubakaran M., Sarada D. V. L. (2013). Evaluation of antioxidant and wound healing potentials of*Sphaeranthus amaranthoides*Burm.f. *BioMed Research International*.

[152] Velíšek J. (2018). Saponins. *Natural Toxic Compounds of Foods*.

[153] Giorno T. B. S., Santos C. H. C. D., Carvalho M. G. D. (2019). Study on the antinociceptive activity and mechanism of action of isolated saponins from siolmatra brasiliensis (Cogn.) Baill. *Molecules*.

[154] Mukherjee P. K., Verpoorte R., Suresh B. (2000). Evaluation of in-vivo wound healing activity of Hypericum patulum (Family: hypericaceae) leaf extract on different wound model in rats. *Journal of Ethnopharmacology*.

[155] Nasri H., Shirzad H. (2013). Toxicity and safety of medicinal plants. *Journal of HerbMed Pharmacology*.

[156] George P. (2011). Concerns regarding the safety and toxicity of medicinal plants- an overview. *Journal of Applied Pharmaceutical Science*.

[157] Kanyoni J., Kenyatta J., Mwangi K. J., Kariuki K. J., Reuben T., Kibe K. G. (2018). The phytochemical components and acute toxicity of methanolic stem bark extract of Prunus africana Experiment Findings · the phytochemical components and acute toxicity of methanolic stem bark extract of Prunus africana. *IOSR Journal Of Pharmacy*.

[158] Karani L. W., Tolo F. M., Karanja S. M., Khayeka C. W. (2013). Safety and efficacy of prunus africana and Warburgia ugandensis against induced asthma in BALB/c mice. *European Journal of Medicinal Plants*.

[159] Gathumbi P. K., Mwangi J. W., Njir S. M., Mugera G. M. (2000). Biochemical and haematological Cha∼Ges in rats administered an aqueous extract of prunus africana stem-bark at various dosage levels. *Onderstepoort Journal of Veterinary Research*.

[160] Gathumbi P. K., Mwangi J. W., Mugera G. M., Njiro S. M. (2002). Toxicity of chloroform extract of Prunus africana stem bark in rats: Gross and histological lesions. *Phytotherapy Research*.

[161] Nabende P. N. (2015). Safety and anti-proliferative activity of Prunus africana, Warburgia stuhlmannii and Maytenus senegalensis extracts in breast and colon cancer cell lines.

